# From mouth to macrophage: mechanisms of innate immune subversion by *Mycobacterium avium* subsp. *paratuberculosis*

**DOI:** 10.1186/1297-9716-45-54

**Published:** 2014-05-15

**Authors:** Ryan J Arsenault, Pekka Maattanen, Joanna Daigle, Andrew Potter, Philip Griebel, Scott Napper

**Affiliations:** 1 United States Department of Agriculture, Agricultural Research Service, SPARC, College Station, TX 77845, USA; 2 VIDO-InterVac, University of Saskatchewan, Saskatoon, SK S7N 5E3, Canada; 3 Department of Biochemistry, University of Saskatchewan, Saskatoon, SK S7N 5E5, Canada; 4 School of Public Health, University of Saskatchewan, Saskatoon, SK S7N 5E5, Canada

## Abstract

Johne’s disease (JD) is a chronic enteric infection of cattle caused by *Mycobacterium avium* subsp. *paratuberculosis* (MAP). The high economic cost and potential zoonotic threat of JD have driven efforts to develop tools and approaches to effectively manage this disease within livestock herds. Efforts to control JD through traditional animal management practices are complicated by MAP’s ability to cause long-term environmental contamination as well as difficulties associated with diagnosis of JD in the pre-clinical stages. As such, there is particular emphasis on the development of an effective vaccine. This is a daunting challenge, in large part due to MAP’s ability to subvert protective host immune responses. Accordingly, there is a priority to understand MAP’s interaction with the bovine host: this may inform rational targets and approaches for therapeutic intervention. Here we review the early host defenses encountered by MAP and the strategies employed by the pathogen to avert or subvert these responses, during the critical period between ingestion and the establishment of persistent infection in macrophages.

## Table of contents

1. Introduction

2. Johne’s disease

3. Species tropisms of MAP

4. Disease transmission

5. Host resistance to MAP infection

6. Zoonotic threat of MAP

7. Stages of MAP infection

7.1 Stage 1: MAP invasion of the intestinal barrier

7.1.1 Tissue uptake of MAP

7.1.2 Mechanisms of MAP invasion from the intestine

7.2 Stage 2: Infection of and survival within the macrophage

7.2.1 MAP invasion of the macrophages

7.2.2 Blocking phagolysosome fusion

7.2.3 Blocking macrophage responsiveness

7.2.3.1 Pattern recognition receptors

7.2.3.2 TLR9

7.2.3.3 TLRs 1 and 2

7.2.3.4 Interferon gamma signaling

7.2.3.5 Superoxide dismutase

7.2.3.6 Nitric oxide

7.2.3.7 Apoptosis

7.2.3.7.1 MAP promotes apoptosis of infected macrophages

7.2.3.7.2 MAP inhibits apoptosis of infected macrophages

7.2.3.8 IL-10

8. Conclusions

9. Abbreviations

10. Competing interests

11. Authors’ contributions

12. References

## 1. Introduction

*Mycobacterium avium* subspecies *paratuberculosis* (MAP) is the causative agent of Johne’s disease (JD), a chronic granulomatous enteritis of cattle. While the characterization of JD in dairy cattle dates back over a hundred years, the challenges and costs imposed by this disease on the livestock industry have increased over time. JD has become more prevalent, hypothesized to result from modern livestock management practices. Furthermore, speculation that MAP may represent a zoonotic threat has elevated the priority of this disease from an issue of food production to one of food safety.

Efforts to control JD through improved animal management efforts have had limited success. This is largely due to difficulties associated with reliable detection of infected animals in the absence of clinical signs of disease, as well as the ability of the pathogen to persist in the environment. These difficulties make traditional approaches to manage the disease largely ineffective, and they place particular emphasis on the need to develop an effective vaccine to prevent disease transmission. To date, the vaccines that have been utilized for JD have reduced MAP shedding and clinical disease but have not been effective in preventing infection. This may further complicate management of the disease by increasing the prevalence of subclinical MAP infections within a herd.

That the vast majority of animals exposed to MAP do not develop clinical disease indicates that the bovine immune system - when appropriately activated - can effectively control the infection. These observations offer guarded confidence that it may be possible to develop a vaccine which can prevent infection. The limited success of vaccine development efforts to date likely reflects the complexity of this host-pathogen interaction; in particular MAPs ability to subvert critical host immune responses. As such, understanding the mechanisms employed by the host as well as the counter-measures employed by the pathogen may reveal rational points of therapeutic intervention.

## 2. Johne’s disease

Clinical manifestations of MAP infection of cattle include diarrhea, progressive weight loss, general wasting and decreased milk production. These clinical symptoms usually appear two to five years after the initial infection, which generally occurs during the neonatal period. Disease progression involves a general deterioration of health and productivity. If the disease is allowed to progress, cattle eventually succumb to either dehydration or cachexia. Notably, in a production setting, infected animals are typically culled shortly after the first indications of clinical disease. Pathology associated with JD is primarily localized to the terminal small intestine but may be much more extensive and encompass both the small and large intestine. The intestinal wall becomes markedly thickened, which may inhibit nutrient absorption, and tissue change is characterized by the extensive formation of submucosal granulomas.

## 3. Species tropisms of MAP

MAP is classically described as a pathogen of ruminants with a host range that includes cattle, sheep, goats, and deer [1,2]. However, MAP has also been isolated from a number of wildlife species including badgers, coyotes, crows, cats, opossums, rabbits and raccoons [3,4]. While the priority of MAP investigations is generally cattle, the ability of this pathogen to infect wildlife species raises concerns that these species could act as disease reservoirs that enable transmission of MAP to naïve livestock herds. Conversely, there is also the threat of disease transmission from MAP-infected domestic herds into the surrounding wildlife.

## 4. Disease transmission

While some calves may be infected with MAP in utero, most infections are thought to occur during the neonatal period. MAP typically enters the calf through the oral route by consuming infected material - soil, feces, MAP infected milk or colostrum - or as a result of oral contact with contaminated surfaces, including udders. MAP may also be transferred by other bodily fluids such as saliva, uterine fluid, or semen [5,6]. Recent studies have indicated that MAP may also be acquired through inhalation of aerosols [7]. Vertical transmission of MAP is most common, however, horizontal transmission from either calf-to-calf or calf-to-contaminated wildlife and contaminated wildlife-to-calf, potentially including insects, has also been observed [8,9]. While wildlife reservoirs of disease represent a potential source of infection, MAP most often enters a cattle herd through the acquisition of an infected cow [9].

The susceptibility of cattle to infection by MAP is largely age-dependent. Calves experience the greatest susceptibility during the first few months of life with increasing tolerance developing between four months to one year [10]. By one year of age, calves achieve a level of MAP resistance comparable to that of an adult cow: they can be infected with MAP but this requires higher infectious doses and longer exposure periods [11,12]. The increased susceptibility of young calves to MAP infection may be due to a number of contributing factors. The higher porosity of the open gut of newborn calves may offer greater opportunity for pinocytosis of MAP [12]. Maternal antibodies to MAP within colostrum may also promote the uptake of MAP through enhanced opsonization of the bacteria [13]. Finally, a general immaturity of the innate and/or adaptive immune systems may contribute to an increased susceptibility of newborn calves to MAP infection [14,15].

## 5. Host resistance to MAP infection

Importantly, only 10-15% of cattle exposed to MAP develop clinical disease, indicating that most calves successfully control the infection [16]. The ability of individual animals to successfully control a MAP infection likely reflects contributing factors such as host health and genetics, environmental conditions, infectious dose and variables associated with different MAP strains. There have been numerous efforts to identify specific host genetic factors that predict MAP susceptibility. Such investigations are typically conducted with the goal of identifying biomarkers to guide the breeding of disease tolerant animals. Unfortunately, as might be anticipated, bovine genetic susceptibility to MAP infection is complex. This is perhaps best exemplified by a recent meta-analysis of two genome-wide association studies that revealed multiple loci associated with MAP infection of cattle involving 11 different chromosomes [17]. The identification of genetic predictors of MAP resistance is further complicated by the breed-specificity of some of these biomarkers. For example, in Brahman-Angus crossbred animals there is an association between infection status and the caspase associated recruitment domain 15 (CARD15) gene that is not observed in Holstein cattle [18]. Other priority resistance genes have been proposed based on i) involvement in other mycobacterial diseases, ii) roles in host defense and iii) associations with Crohn’s disease, including solute carrier family 11 member 1 (SLC11A1) (also known as NRAMP1), Toll-like receptors (TLRs), major histocompatibility complex (MHC) and cytokines (interleukin-10 and interferon-gamma) and their receptors [19].

## 6. Zoonotic threat of MAP

The economics costs, as well as animal health and welfare considerations, are sufficient to define JD as a priority for the livestock industry. However, the disease has taken on even greater importance with the possible implication of MAP as a causative agent or contributing factor in Crohn’s disease, an inflammatory bowel disease of humans [20].

In the absence of definitive evidence regarding the contribution of MAP to Crohn’s disease, mitigating the risk of MAP transfer from cattle to humans may be an important precautionary action. Reducing MAP transmission in cattle would not only have a significant positive impact on public perception, reducing MAP transmission in cattle would have direct economic benefits to livestock producers. By themselves, animal health and welfare aspects of JD are of sufficient importance to justify the development and implementation of a vaccination program that could prevent transmission of MAP. Historically, vaccination has been an extremely effective tool for control of infectious diseases in humans and animals. Unfortunately efforts to develop an effective vaccine for MAP have had limited success [21]. While the initial BCG vaccine developed in the early 1900s is still utilized, the functional utility of this vaccine is largely limited to reduced shedding of MAP by infected animals [22]. Mycopar® (strain 18), a whole inactivated MAP vaccine has only shown protection from clinical disease, but not infection, potentially exacerbating the problem of asymptomatic carriers [22]. The limited success of traditional approaches of vaccine development for JD indicates that a greater understanding of the virulence mechanisms of MAP is required in order to adopt a more strategic approach to vaccine design. Specifically, we propose that understanding the mechanisms by which MAP subverts host immune responses will form the basis for developing and evaluating an effective vaccine.

## 7. Stages of MAP infection

The primary focus of this review is to critically review what is known regarding events that occur during the earliest stages of MAP infection of the bovine host. Specifically the host defenses activated and bacterial counter-measures employed during the period between oral uptake of MAP and its establishment of a persistent infection within macrophages. This critical period can be sub-divided into two distinct functional stages: 1) MAP invasion of the gut, and 2) Infection and survival in macrophages. Within each stage MAP encounters a variety of host defense systems that must be averted or subverted in order for the pathogen to establish infection.

### 7.1 Stage 1: MAP invasion of the intestinal barrier

#### 7.1.1 Tissue uptake of MAP

MAPs' efforts to establish infection are not limited to considerations of the conditions that exist within a single animal; they extend to changes in conditions that exist across hosts. Within infected herds horizontal transmission of MAP often occurs during calves’ nursing from an infected dam. Patel et al. demonstrated that pre-incubation of MAP in mammary epithelial cells or milk increased MAPs ability to infect bovine epithelial cells, suggesting that the cellular environment within the infected host primes the pathogen for invasion of intestinal epithelial cells of the next target host [13]. Gene expression analysis attributed this increased virulence to a small subset of genes which included virulence factors and MAP-specific genes of unknown function [23].

**Figure 1:**
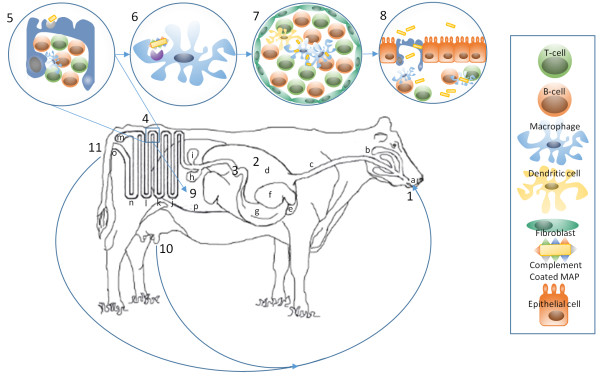
**Uptake of orally ingested MAP.** MAP is ingested (1) and travels through the GI tract. It may also be taken up in tonsillar crypts and transported to the ileum. In the rumen (2) the bacterium’s FAP is activated and is opsonized by fibronectin upon entering the lower digestive tract (3). After reaching the ileum (4) MAP is phagocytized by M cells of Peyer’s patches following recognition of the bacterium through the fibronectin receptor (5) and travels across the epithelium to intra-epithelial macrophages, which take up complement-coated MAP via complement receptors (6). Infected macrophages form granulomas (7), which harbor latent MAP infections. During active JD (8), MAP may be transmitted to an unborn calf (5), to neonates following priming in mammary glands and in milk causing increased virulence (9), or through fecal matter contaminating the environment (10). (a) Mouth (b) Salivary Glands (c) Esophagus (d) Rumen (e) Reticulum (f) Omasum (g) Abomasum (h) Gallbladder (i) Pancreas (j) Duodenum (k) Jejunum (l) Ileum (m) Cecum (n) Large Intestine (o) Anus (p) Uterus.

Oral challenge models suggest MAP may invade the host as early as the oral mucosa in the tonsils. This conclusion is supported by models that have demonstrated the tonsillar crypts as a route for MAP infection [24]. Following entry through the tonsils it is proposed that MAP spreads to the mesenteric lymph nodes and ileum via the blood [25]. There is some concern, however, over the physiological significance of these routes of infection. Less aggressive oral infection models, using lower doses of MAP, have implicated the ileum as the primary point of MAP invasion [25] (Figure 1).

Using a gut surgical loop model, our group observed that MAP infected the jejunal region of newborn calves with the induction of strong, but non-fatal, mucosal immune responses. MAP infection of the ileal region of newborn calves was also observed within our intestinal loop infection studies [26,27]. Following oral ingestion of MAP, while all regions of the small intestine should be exposed to bacteria, there is increasing evidence for significant regional differences in mucosal immune system development during the neonatal period [14,15]. Further characterization of innate and acquired immune responses following MAP infection at specific sites throughout the gastro-intestinal tract may be of considerable significance in understanding MAP pathogenesis. It will be important to determine if the ileal Peyer’s patch (PP) truly provides a unique portal of entry by which MAP establishes persistent infections. There are marked age-dependent changes in the structure and function of this lymphoid tissue [28] which may be consistent with an age-dependent susceptibility to MAP infection. Furthermore, this information may also be critical for the design and delivery of a protective vaccine if immune effector cells must localize to a specific region of the small intestine.

#### 7.1.2 Mechanisms of MAP invasion from the intestine

Experimental infection studies have suggested that MAP invasion appears to occur through both microfold (M) cells of the Peyer’s patches as well as differentiated epithelial cells [29-31]. Passage of MAP through the ruminant digestive system activates the bacterial cell wall protein fibronectin attachment protein (FAP) to promote opsonization by fibronectin [32]. Fibronectin, in turn, links MAP to the luminal surface of intestinal M cells through fibronectin receptors. This fibronectin-dependent mechanism of MAP uptake likely contributes to the preferential uptake of MAP through M cells, which are enriched with the β1 fibronectin receptor [33]. There may be alternate, fibronectin-independent, mechanisms for MAP entry into intestinal epithelial cells, although the physiological significance of these routes of entry has yet to be firmly established [31]. The MAP invasion of this first host cell barrier occurs very quickly, within 30 min of contact [34]. These cells then translocate MAP from the intestinal lumen to the submucosa [35]. Within the submucosa MAP is then ingested by macrophages. Following an experimental infection, MAP translocation from the intestinal lumen into submucosal macrophages occurs in a matter of minutes to hours [35] (Figure 1).

### 7.2 Stage 2: Infection of and survival within the macrophage

Macrophages play a critical role in the host-pathogen interaction of JD. Not only do they serve as a central effector for mediating the destruction of MAP but also, in the event of their subversion by MAP, being transformed into protected havens for the survival, proliferation and dissemination of the pathogen.

#### 7.2.1 MAP invasion of the macrophages

After penetrating the intestinal epithelial barrier, MAP specifically invades sub-epithelial macrophages, which have several families of receptors involved in the uptake of mycobacteria: the complement receptors (CR1, CR3, and CR4), the immunoglobulin receptors (FcR), the mannose receptor and scavenger receptors [36]. Different routes of entry into macrophages may have important consequences for intracellular survival, each utilizing different receptor-mediated systems and resulting in unique patterns of cytokine secretion inducing differential immune responses [36]. In particular, uptake via complement receptors limits macrophage activation [37]. That the uptake of MAP into bovine macrophages is enhanced by the opsonization with the serum from either healthy or JD cattle indicates complement-mediated uptake of MAP [38]. Preferential uptake of MAP into bovine macrophages through the complement system may represent a strategy of the bacteria to evade critical host defenses (Figure 1).

#### 7.2.2 Blocking phagolysosome fusion

One of the most critical periods in the establishment of a persistent infection occurs immediately after MAP entry into the macrophage phagosome. Through programmed changes in membrane markers and acidification of the internal compartment, phagosomes undergo step-wise development to a late endosome. Fusion of the late endosomes with lysosomes, creating phagolysosomes, generates an environment that is chemically and biochemically tailored for the destruction of internalized particles, including MAP [39]. The survival of the internalized bacteria depends on its ability to disrupt the formation of the mature phagolysosome in order to avoid the resulting hydrolysis and oxidation reactions. Inhibition of phagosome acidification and phagolysosome fusion represent critical mechanisms by which mycobacteria survive within macrophages [40].

Macrophage subversion requires strategies of active intervention by mycobacterial pathogens. Cheville et al. demonstrated changes in patterns of endosomal markers that supported the ability of live, but not dead, MAP to block endosomal maturation [41]. Within the same study, a similar trend was observed whereby only viable MAP was able to block the acidification of phagosomes [41]. Investigations performed by other groups, considering other markers of phagosomal maturation, such as the Rab GTPase family, have reached similar conclusions that live MAP is required for inhibition of phagosomal maturation [40].

**Figure 2:**
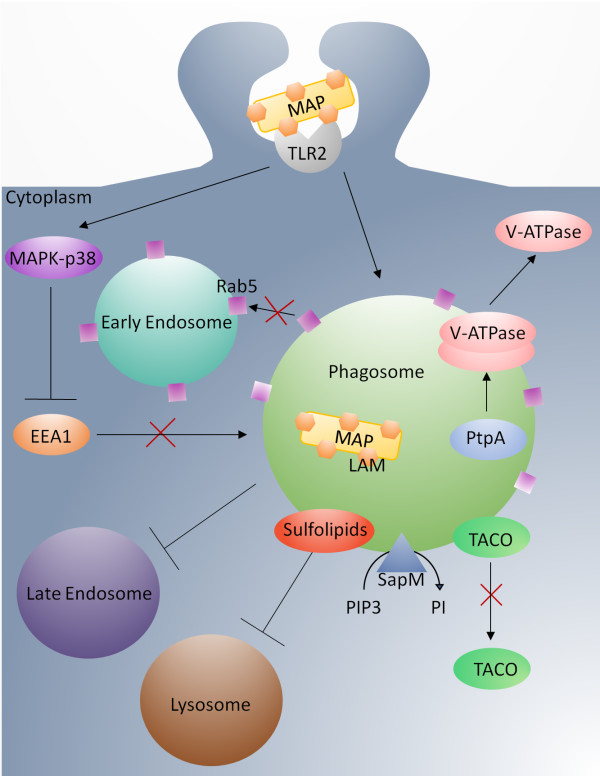
**Inhibition of phagolysosomal maturation by MAP.** MAP sulpholipds inhibit the formation of the phagolysosome by hindering the merging of the phagosome with the lysosome. SapM dephosphorylates phosphotidylinositol phosphates, disrupting membrane-trafficking regulation. V-ATPase is involved in phagosome-lysosome fusion. It is bound by mycobacterium protein PtpA and excluded from the phagosome thus inhibiting fusion. Rab5 stimulates fusion of early endosomes. Through retention of Rab5, as well as inhibition of recruitment of early endosomal autoantigen 1 (EEA1) to mycobacterial phagosomes, MAP is able to avert the maturation of endosomes into functional mycobacteriocidal compartments. Normally TACO is released from the phagosome allowing the lysosome to fuse. This release is inhibited by MAP. Mycobacteria are known to influence MAPK-p38 through LAM activation of TLR2. This ultimately leads to the inhibition of EEA1. TLR2 also induces production of IL-10 inhibiting a number of other innate immune signaling pathways.

Given the importance of phagolysosomal maturation, it is perhaps not surprising that MAP employs a number of complementary - and potentially redundant - mechanisms for subversion of this process. In one scenario, inhibition of phagosome maturation and phagosome-lysosome fusion is mediated through MAP’s efforts, directed on the phagosomal membranes. For example, mycobacterial lipids are proposed to integrate into - and thereby disrupt the structural and functional characteristics of the phagosome membranes [42]. Specifically, a mycobacterial sulfolipid has been shown to be sufficient to impair phagosome fusion [43]. Mycobacteria can also influence the functional characteristics of the phagosome membrane through the secretion of the lipid phosphatase SapM. During phagosome development, phosphatidylinositol 3-phosphate (PI3P) is an essential membrane-trafficking regulatory lipid that allows the phagosome to acquire lysosomal constituents [44]. In host cells infected with dead mycobacteria, PI3P functions normally, but in host cells infected with live mycobacteria, PI3P is eliminated on a continuous basis by the hydrolysis activity of SapM [40]. This mechanism is likely used by MAP as, based on our NCBI-NR Blast results, its genome contains a phosphoesterase protein very similar in amino acid sequence to the SapM found in *M. tuberculosis* (Figure 2).

Acidification of the phagosome is an essential component of the maturation process as lysosomal vacuoles contain a complement of hydrolytic enzymes that depend on an acidic environment for their optimum catabolic activity. Vacuole acidification is achieved through the action of the vacuolar H^+^ − ATPase (V-ATPase), a primary active transport protein that utilizes the energy of ATP to pump protons into the vacuole [45]. Disruption of vacuole acidification is a priority evasion mechanism for intracellular pathogens; phagosomes containing pathogenic mycobacteria have substantially higher pH than those containing either nonpathogenic or killed organisms [45]. Through neutralization of this pump or inhibition of its recruitment to the vacuole MAP is able to prevent phagosomal acidification [46]. Through gene expression studies Weiss et al. and Murphy et al. [47,48] found that expression of the V-ATPase was higher in MAP-infected macrophages as compared to those infected with non-pathogenic mycobacteria [49]. These findings highlight this system’s priority to MAP infection and indicate MAP’s actions are not mediated at the level of regulation of transcription. Instead MAP secretes a protein effector molecule, protein tyrosine phosphatase (PtpA), into the vesicle contents. PtpA binds a specific subunit of the macrophage V-ATPase machinery that is responsible for luminal acidification and is speculated to coordinate phagosome-lysosome fusion through interaction with the macrophage class C vacuolar protein sorting complex. PtpA, through its interaction with the V-ATPase, mediates dephosphorylation of VPS33B resulting in exclusion of V-ATPase from the phagosome thereby inhibiting phagosome acidification [50] (Figure 2).

Maturation of the endosomes occurs in a stepwise, highly regulated fashion. A number of host proteins, including ATPase N-ethylmaleimide- sensitive factor (NSF), soluble NSF attachment proteins (SNAPs), and vesicle and target membrane SNAP receptors (SNARES) are essential for the maturation and fusion of endosomes [51,52]. These processes are regulated through Rab GTPases; Rab5 stimulates fusion of early endosomes while Rab7 promotes fusion of mature phagosomes with endosomes and lysosomes [53]. By retaining Rab5 and inhibiting recruitment of early endosomal autoantigen 1 (EEA1) to mycobacterial phagosomes, MAP is able to avert the endosome’s maturation into functional mycobacteriocidal compartments [54] (Figure 2). Additional mechanisms for MAP’s inhibition of phagolysosome membrane fusion have been described. One such mechanism may be halting the release of the tryptophan aspartate-containing coat protein (TACO) from the phagosome. Normally, TACO is released from the phagosome, allowing fusion with the lysosome [55] (Figure 2).

Mycobacteria also appear to subvert the maturation of the endosomes through higher-order influences on cell signaling. Studies have demonstrated interconnection between mitogen activated protein kinase (MAPK)-p38 signaling and the endocytic pathway. Specifically, MAPK-p38 signaling blocks the association of EEA1 and LAMP-3 with late endosomes, whereas blocking MAPK-p38 signaling causes phagosomal acidification and enrichment of the late endocytic markers [56]. This suggests that MAPK-p38 has a negative regulatory role in phagolysosome biogenesis (Figure 2).

#### 7.2.3 Blocking macrophage responsiveness

##### 7.2.3.1 Pattern recognition receptors

Pattern recognition receptors (PRRs) are responsible for the perception of microbial challenge with subsequent induction of protective host responses [29]. Activation of PRRs occurs through the binding of receptor-specific pathogen associated molecular patterns (PAMPs). The Toll-like receptors (TLRs) represent a major PRR sub-category. In addition to the standard immune induction and cytokine responses initiated by the various TLRs, other cellular processes important to mycobacterial pathogenesis are also TLR-mediated, including phagosomal maturation [57]. Individuals with mutations in the TLR system show increased susceptibility to a variety of infectious challenges, including mycobacteria [58]. Further, dynamic levels of TLR expression in response to natural MAP infection implicate a role for this system in protection against MAP. The specific role of the TLR system in defense against MAP appears complex. Activation of certain TLRs, such as TLR9, seem to initiate responses that are critical in defense against MAP [59] while activation of other TLRs, such as TLR2, induce responses that suppress immune defense against MAP [60].

##### 7.2.3.2 TLR9

While several mammalian TLRs are involved in controlling mycobacterial infection [61,62] TLR9 is of particular interest due to its role in natural defense as well as the potential for use of TLR9 agonists as immunotherapeutics for mycobacterial infections [59]. TLR9 recognizes and binds to microbial DNA as identified by the presence of unmethylated CpG motifs. In mouse models, CpG oligodeoxynucleotides (ODNs) have value in the treatment of mycobacterial infections [63] and pretreatment of human macrophages with CpG ODNs, but not other TLR ligands, promoted phagolysosome development and inhibited *M. tuberculosis* growth [64].

**Figure 3:**
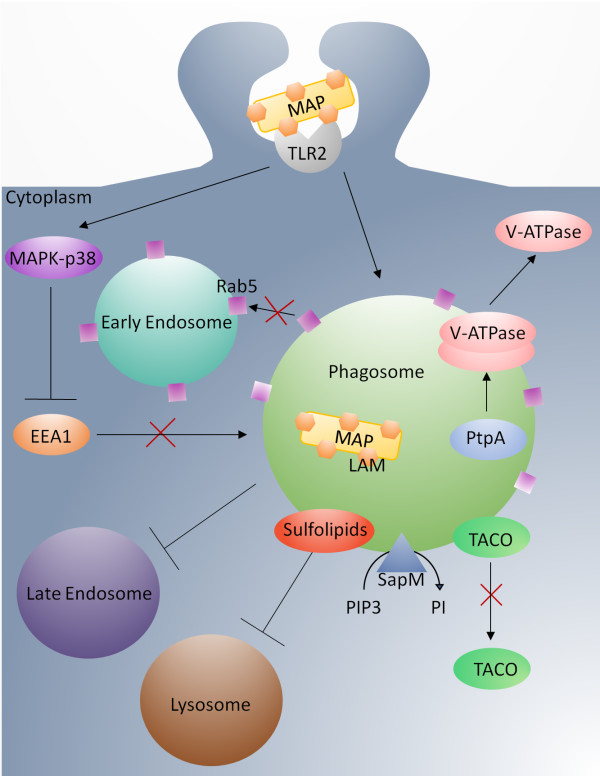
**Inhibition of toll-like receptor signaling by MAP.** Signaling pathways based on known phosphorylation events of TLR9. A coloring scheme is used to illustrate phosphorylation events that were detected by peptide array kinome analysis when analyzing lysates from bovine monocytes stimulated with CpG ODNs in the presence and absence of MAP infection. Differential levels of phosphorylation relative to the media treated control (*p* < 0.1) are presented. Green for increased phosphorylation, red for decreased phosphorylation and blue for insignificant phosphorylation or peptide not present on array. Adapted from Arsenault et al. [89]. (Copyright © American Society for Microbiology).

Given the demonstrated role of TLRs, specifically TLR9, in host defense against MAP, as well as the therapeutic potential of TLR9 agonists in the treatment of mycobacterial infections, our group investigated the consequences of MAP infection on the responsiveness of bovine monocytes to TLR9 stimulation [65]. Inspite of a 10-fold increase in TLR9 expression and maintained uptake of CpG ODNs, classical TLR9-mediated responses were inhibited in MAP-infected bovine monocytes. Other TLR9-mediated responses, which occur through non-canonical signaling, including oxidative burst, remained functional. Kinome analysis using species-specific peptide arrays confirmed that classic TLR9 signaling is redirected by MAP to proceed through a Pyk2 mediated mechanism (Figure 3). Pyk2 mediated signaling does not hinder infection as CpG ODNs fail to promote MAP clearance. Instead Pyk2 signaling appears to function to the advantage of the pathogen: treatment of MAP-infected bovine monocytes with Pyk2 inhibitors significantly reduced the number of intracellular MAP bacteria [65]. Although the mechanism by which MAP exerts this nuanced effect on TLR9 signaling is unknown, these results are consistent with a multifaceted strategy to generate an intracellular environment that favors a persistent infection.

##### 7.2.3.3 TLRs 1 and 2

Toll-like receptor 2 (TLR2), through the formation of heterodimers with TLRs 1 and 6, has the potential to bind to a range of PAMPs [66]. Polymorphisms of TLR2 have been associated with increased susceptibility of cattle to paratuberculosis [58]. TLR2 has been implicated in the recognition of mycobacteria through the binding of bacterium cell wall lipoproteins [18]. TLR2 is activated by Mannosylated liparabinomannan (ManLAM) to initiate signaling though the MAPK–p38 pathway. A primary outcome of this signaling is increased interleukin (IL)-10 gene expression. IL-10 is known to suppress pro-inflammatory cytokines, chemokines, IL-12, and major histocompatibility factor class-II expression. The induction of IL-10 expression through Man-LAM-induced TLR2-MAPK-p38 signaling is a primary mechanism by which MAP suppresses the antimicrobial responses of bovine macrophages. Indeed, many of the suppressive effects of MAP on bovine macrophages can be replicated by exposing bovine monocytes to purified Man-LAM. The subversive effects of MAP on infected macrophages can, conversely, be reversed by pre-incubating cells with neutralizing anti-TLR2 antibodies. This pre-treatment results in increased phagosome acidification, phagosome maturation, and the killing of MAP [67]. That anti-TLR2 antibody treatment does not decrease the IL-10 concentration in culture supernatants indicates that TLR2 has IL-10-independent roles in altering phagosome acidification and maturation, such as through inhibition of EEA1 (Figure 2).

ManLAM also reduces host gene expression of tumor necrosis factor alpha (TNFα) and IL-12. It also increases the expression of Src homology region 2 domain-containing phosphase-1 (SHP-1) [68]. SHP-1 is a tyrosine phosphatase that down-regulates macrophage immune responses. ManLAM has also been found to inhibit the expression of IL-12 in host dendritic cells [69]. *M. tuberculosis* and *M. bovis* bacilli Calmette-Guérin are two *Mycobacterium* species that also contain ManLAM in their cell wall. Exposure of host cells to ManLAM from these species resulted in no detectable TLR-dependent immune activation [70]. ManLAM has been implicated in the inhibition of interferon gamma (IFNγ)-induced macrophage activation [71] and the induction of transforming growth factor beta (TGFβ) [72]. The elimination of IFNγ and the activation of TGFβ pathways can lead to a switch from a Type 1 helper T-cell (Th1) to a Th2 immune response, which is generally considered ineffective against intracellular mycobacterial infections [72].

From various studies, it appears that TLR1 and TLR2 are affected differently by mycobacterial infection. In both mesenteric lymph node (MLN) mononuclear cells and in peripheral blood mononuclear cells (PBMCs) from cattle infected with MAP a significant decrease in TLR1 expression is observed. In the same studies, there was no significant change in the expression of TLR2 [62]. Thus the immune effects may be the result of a lack of subsequent interaction between TLR1 and TLR2.

##### 7.2.3.4 Interferon gamma signaling

IFNγ plays a central role in immune defense against a variety of intracellular pathogens, including mycobacteria [73]. IFNγ signaling induces a range of bactericidal responses including induction of reactive oxygen and nitrogen intermediates, production of cytokines and promotion of phagosome maturation. IFNγ activates target cells by binding to a high-affinity IFNγ receptor for activation of the Janus family kinase - Signal Transducer and Activator of Transcription (JAK-STAT) pathway [74]. Mice deficient in IFNγ are more susceptible to intracellular pathogens [75]. Humans with mutations in the IFNγ receptor chains experience infection by low-virulence environmental mycobacterial strain infections and suffer from recurring bouts of tuberculosis [76]. Multiple studies have outlined the importance of IFNγ to the pathogenesis of JD. Cattle in the subclinical, excretory stage of JD produce increased levels of IFNγ at the ileal and cecal lymph nodes [77]. When PBMCs are isolated from infected cattle and stimulated with MAP antigens in vitro, they display a greater release of IFNγ than that observed in cells from uninfected cattle [78]. Furthermore, we observed increased MAP-specific IFNγ production by mesenteric Ln cells within one month after MAP infection [27]. Therefore, IFNγ production appears to be an early response to MAP infection and this response continues throughout a persistent infection.

Given the importance of IFNγ for control and clearance of intracellular pathogens, this host defense mechanism is a logical target for disruption by MAP. A number of other pathogens have been shown to suppress IFNγ and JAK/STAT1/2 signaling through a number of mechanisms including decreased expression of IFNγ receptors [79,80], decreased association of STAT with transcriptional co-activators [79], and induced expression of suppressor of cytokine signaling (SOCS), which binds and inactivates JAK to block JAK/STAT signaling [80]. Man-LAM also promotes activation of tyrosine phosphatase-1, an inhibitor of JAK/STAT signaling [68]. The clinical observation that IFNγ production persists throughout MAP infection suggests that the evasion strategy used by MAP is disruption of IFNγ signaling. Specifically, while pretreatment of macrophages with IFNγ enhanced their ability to clear mycobacterial infections [81] the same treatment, given post-infection, is unable to achieve efficient clearance of the bacterium [82]. These results suggest that once MAP has established itself with the macrophage it is able to inhibit IFNγ dependent clearance; however, if the antimicrobial activity of the macrophage is stimulated by IFNγ prior to infection, clearance is possible.

**Figure 4:**
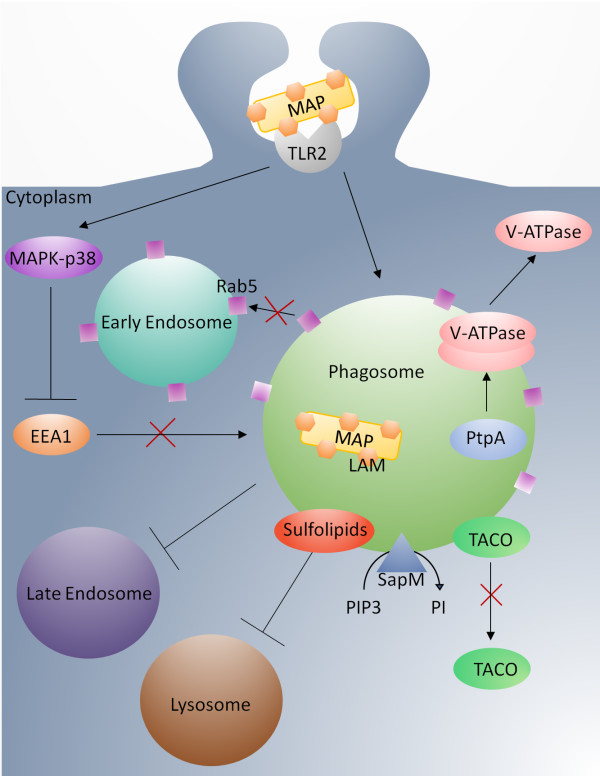
**Inhibition of IFNg signaling by MAP.** MAP infection inhibits JAK-STAT signaling via increased expression of negative regulators of the IFNγ receptor, SOCS1 and SOCS3, as well as decreased expression of IFNγ receptor chains 1 and 2. These modifications of cellular responsiveness occurred in temporal fashion with early induction of SOCS1 and 3 with subsequent repression of the IFNγ receptor. MAP inhibition of IFNγ responsiveness may be mediated by MAP effector molecule PtpB. PtpB may disrupt the normal IFNγ receptor immune signaling pathway by dephosphorylating a key signaling intermediate, or perhaps the receptor itself.

To investigate the mechanism (s) by which MAP blocks IFNγ-dependent responses our group used kinome analysis to compare monocyte responses to IFNγ in the presence or absence MAP infection [65]. Significant JAK-STAT signaling was observed in IFNγ-stimulated bovine monocytes in the absence of MAP infection. In contrast, IFNγ stimulation of MAP-infected bovine monocytes failed to induce patterns of peptide phosphorylation consistent with JAK-STAT activation. The inability of exogenous IFNγ to induce differential phosphorylation of peptides corresponding to early JAK-STAT intermediates in infected monocytes suggested that MAP blocked signaling at, or near, the IFNγ receptor. Further investigation revealed that following MAP infection there was increased expression of SOCS1 and SOCS3 genes, negative regulators of the IFNγ receptor, as well as decreased expression of IFNγ receptor chains 1 and 2 genes. These transcriptional changes occurred sequentially with early induction of SOCS1 and 3 genes and subsequent repression of the IFNγ receptor genes [65] (Figure 4). These observations agree with previous findings that MAP infection of mouse macrophages inhibited JAK-STAT signaling via decreased expression of the IFNγ receptor genes [83].

It has been suggested that MAP inhibition of IFNγ responsiveness is mediated by the specific effector molecule PtpB. *M. tuberculosis* with the mPtpB gene knocked-out survived as well as wildtype bacteria within unstimulated macrophages, but survival of the mutant mycobacterium was severely impaired when infected macrophages were stimulated with IFNγ [84]. These observations support the conclusion that PtpB plays an essential role in disrupting the IFNγ receptor signaling pathway by either dephosphorylating a key signaling intermediate or the receptor itself. MAP may employ more than one mechanism to evade IFNγ induced antimicrobial responses since purified sulpholipids from mycobacteria have also been found to block immune priming of human monocytes as indicated by the failure of these cells to induce superoxide production in response to IFNγ stimulation [85] (Figure 4).

Thus, if MAP establishes infection in quiescent macrophages it can alter the expression of IFNγ receptors and activate pathway suppressors to ensure evasion of both innate and acquired immune responses. Understanding these evasion mechanisms and the cells within which they are active may contribute to the rational design of a protective vaccine and/or therapeutics that promote clearance of a MAP infection. Developing vaccines that induce a strong Th1 or IFNγ response may be inadequate if these responses are unable to activate IFNγ-induced antimicrobial responses prior to MAP infection. Therefore, vaccination prior to MAP exposure is a major challenge when newborn calves are the primary population at risk.

##### 7.2.3.5 Superoxide dismutase

The bactericidal activity of the mature phagolysome is enhanced through the production of reactive chemical molecules, including reactive oxygen intermediates such as superoxide anion, hydrogen peroxide and hydroxyl free radicals [42]. While these reactive species are associated with the killing of mycobacteria [86] there is controversy over the extent to which ROIs contribute to the destruction of intracellular MAP. Specifically, while bovine monocytes generate superoxide anion in response to stimulation with a number of stimulants, very little ROI is produced in response to MAP infection or to IFN-γ stimulation of MAP infected monocytes [87]. These observations may be interpreted as evidence that ROIs are ineffective against MAP and therefore are not activated by the host. Conversely, ROIs may represent a significant threat to MAP and therefore MAP has evolved to subvert an important host defense. MAP has the capacity to secrete superoxide dismutase, which neutralizes superoxide radicals which suggests there may be an advantage in neutralizing this host defense [88]. Inhibition of ROI production may also occur as a consequence of MAP inhibition of IFN-γ induced signaling [65]. Interestingly, the inhibition of TLR9 signaling by MAP does not result in an inhibition of oxidative burst [89].

##### 7.2.3.6 Nitric oxide

The harsh environment of the phagolysosome includes production of another group of anti-mycobacterial molecules, the reactive nitrogen intermediates (RNIs) [90]. Similar to the ROIs there is controversy as to the contribution of RNIs to host defense against different mycobacteria in different species. In mouse macrophages, the IFN-γ-induced production of nitric oxide is directly related to the ability to kill a number of mycobacteria including *M. tuberculosis*, MAP, and *M. leprae*[71,82,90]. Further, chemical inhibition of nitric oxide production increases the intracellular survival of *M. tuberculosis* in mouse macrophages [90]. The relevance of RNIs to JD is supported by the ability of MAP-infected bovine monocytes to increase nitric oxide production in response to IFN-γ stimulation [91]. While chemically-generated nitric oxide is effective in killing MAP the quantities of nitric oxide that are produced in bovine mononuclear phagocytes are generally considered insufficient to mediate exclusive destruction of the bacteria [91]. That mycobacteria inhibit the recruitment of nitric oxide synthase to mycobacteria-containing phagosomes does suggest that this host defense represents a potential threat to survival of this pathogen [92].

##### 7.2.3.7 Apoptosis

There is general consensus that MAP influences the apoptotic tendencies of bovine macrophages, but with conflicting opinion on whether this is in a pro- or anti- apoptotic manner. However, it may be inappropriate to classify MAP as either pro- or anti-apoptotic. Instead, under different situations, or at different stages of the infection cycle, MAP may assume either of these roles. For example, virulent strains of *M. tuberculosis* have been shown to initially postpone apoptosis to allow early intracellular replication, and later induce necrotic cell death to exit the cell when intracellular conditions no longer favor growth [93].

From the perspective of the host, apoptosis of infected cells serves to clear the pathogen. Apoptosis, but not necrotic macrophage death, of mycobacteria-infected macrophages induces the intracellular killing of bacilli. Further, MAP infected immune cells that undergo apoptosis are phagocytosed by healthy macrophages, providing the host immune system opportunity to limit further MAP growth [94]. However, this process may be a means of spreading to healthy macrophages while remaining hidden from immune surveillance, limiting the inflammatory response. Apoptosis of infected macrophages also provides the opportunity for presentation of MAP antigens to guide adaptive immune responses through efferocytosis, a process associated with macrophages and neutrophils, distinct from Fc receptor-mediated apoptosis [95]. Apoptosis of MAP-infected macrophages also limits the acute inflammation and tissue damage that typically occurs with the release of chemotactic molecules following cell lysis.

From the perspective of MAP, establishing a persistent infection depends on its ability to infect new macrophages prior to activation of their antimicrobial defenses. Thus, the local microenvironment into which MAP is released from infected host cells may be critical. MAP release from apoptotic cells or the phagocytosis of MAP-infected apoptotic bodies may be one mechanism to avoid inflammation and activation of uninfected macrophages. A protected environment within the macrophages provides an ideal haven for replication but to maintain a persistent infection it is critical that adjacent macrophages also provide an appropriate site for further replication.

##### 7.2.3.7.1 MAP promotes apoptosis of infected macrophages

Several investigations indicate that MAP infection of macrophages promotes apoptosis [96]. This appears to be an active effort by the bacteria, or the result of a macrophage response to live bacteria, as heat-killed MAP induce significantly less apoptosis [96]. Periasamy et al. reported no change in macrophage death at low MAP MOIs (MOI = 1) but caspase-dependent apoptosis was observed at higher MOIs (MOI = 10) with caspase- and nitric oxide-independent apoptosis and necrosis at the highest MOIs (MOI = 50). These observations were interpreted as evidence that mitochondrial damage initiates cell death processes in MAP infected macrophages [97]. A change in the mechanisms mediating apoptosis at different MOIs may parallel events that occur during natural infection.

##### 7.2.3.7.2 MAP inhibits apoptosis of infected macrophages

Another school of thought suggests that suppression of macrophage apoptosis may be central to the immune evasion strategy employed by MAP and other mycobacterium. Infected macrophages undergo apoptosis for a number of reasons, one of which may be to facilitate more efficient presentation of bacterial antigens to the immune system [98]. Thus, MAP may inhibit apoptosis to not only allow more time for bacterial replication but to also decrease detection by the immune system. MAP-infected macrophages are more resistant to H_2_O_2_-induced apoptosis, normally an inducer of macrophage apoptosis [98]. One mechanism by which MAP inhibits apoptosis is by reducing expression of capase 3/7 and 8 genes resulting in decreased caspase 3/7, 8, and 9 activity [99]. Additionally, by inducing macrophage secretion of IL-10, pathogenic mycobacteria may also limit apoptosis since IL-10 inhibits TNFα expression and increases release of soluble TNFR2 to neutralize TNFα activity [39]. Man-LAM has also been implicated in the regulation of apoptosis by preventing an increase in cytosolic calcium concentration. Cytosolic calcium facilitates apoptosis by increasing mitochondrial membrane permeability, resulting in the release of pro-apoptotic products [39]. Man-LAM also stimulates phosphorylation of Bad a pro-apoptotic protein which prevents the molecule from binding to anti-apoptotic proteins, such as Bcl-2. Free Bcl-2 prevents release of cytochrome c from mitochondria [30]. Interestingly, recent kinome analysis of MAP-infected intestinal tissues identified an over-representation of pro-survival signaling [27]. The pro-survival signaling in the MAP infected tissue was most pronounced in animals developing early antibody responses to MAP antigens but not the typical cell-mediated immune responses associated with MAP clearance.

##### 7.2.3.8 IL-10

The cytokine IL-10 is produced by monocytes, macrophages, and B- and T- lymphocytes [99]. IL-10 reduces expression of IL-12, thus, suppressing the Th1-type immune responses [100]. IL-10 also inhibits production of pro-inflammatory cytokines and decreases antigen presentation by macrophages and dendritic cells [99]. All of these effects, as discussed elsewhere in this review, have the potential to enhance MAP survival within macrophages. Weiss et al. reported that MAP infection resulted in an even greater expression of IL-10 than the closely related bacterial species *Mycobacterium avium* subsp. *avium*[47]. In a subsequent study, neutralizing the IL-10 produced by macrophages infected with MAP there was an increase in: expression of TNFα, IL-12, IL-8, MHC-II; acidification of phagosomes; apoptosis of macrophages; and production of nitric oxide [99]. Each of the above is known to enhance the immune killing of MAP; and indeed, 57% of MAP bacteria were killed within 96 h post-infection with IL-10 neutralization. Understanding the mechanism (s) by which MAP enhances macrophage secretion of IL-10 will provide insight into the immune evasion strategies that contribute to a persistent infection. This information may also provide the rational basis for developing an attenuated vaccine strain that induces protective immunity without the risk of a persistent infection.

## 8. Conclusions

MAP is a pathogen of clear concern for animal health and it results in a significant economic cost to dairy and other livestock producers. To date, limited success has been shown in controlling, treating and ultimately curing MAP infection. The losses incurred by the dairy industry due to infected animals, and the ongoing concerns of a zoonotic disease threat through contaminated dairy products, suggest that a successful vaccine against MAP infection would be a valuable and welcome development. Due to the immune evasion strategies employed by MAP to subvert both the induction of acquired immune responses and immune effector responses, a vaccine that prevents MAP infection remains elusive. The crucial period for intervention in a MAP infection is before or during MAP invasion and establishment of infection within the macrophage. This is the stage we have reviewed here. MAP is very effectively shielded from the host immune system once it is established and growing within the macrophage. To be successful in producing a specific therapeutic or vaccine for a bacterium such as MAP one must understand the mechanisms of immune evasion and pathogenesis. We have discussed how MAP invades the mucosal barrier, and the mechanisms employed to establish a persistent infection. Several strategies involving specific bacterial components are employed in concert by MAP to allow successful host colonization. Any of these bacterial components, or a combination, may be useful targets for therapeutic intervention or vaccine generation. The knowledge of MAP that we have reviewed here has been generated by host-pathogen interaction studies, genomics, kinomics and proteomics. These detailed studies of the mechanism of MAP pathogenesis will hopefully lead to the targeted interventions that are necessary for the treatment of MAP. Ultimately this will enhance animal health, animal production and human food safety.

## Abbreviations

ASK1: Apoptosis signal-regulating kinase 1; AP-1-Jun: Activator protein-1-JUN; ATP: Adenosine triphosphate; BCG: Bacillus Calmette-Guerin; Bcl-2: B-cell lymphoma 2; CARD15: Caspase associated recruitment domain 15; CD4+: Cluster of Differentiation 4 positive; CpG: Cytosine phosphodiester guanine; CR1: Complement receptor 1; CR3: Complement receptor 3; CR4: Complement receptor 4; DNA: Deoxyribonucleic acid; EEA1: Early endosomal autoantigen 1; Erk1: Extracellular signal-regulated kinase 1; Erk2: Extracellular signal-regulated kinase 2; FADD: Fas-Associated protein with Death Domain; FAP: Fibronectin attachment protein; FcR: Immunoglobulin receptors; GI: Gastrointestinal; GTP: Guanidine triphosphate; IFNγ: Interferon gamma; IκBα: Nuclear factor of kappa light polypeptide gene enhancer in B-cells inhibitor, alpha; Ikkα: Inhibitor of nuclear factor kappa-B kinase subunit alpha; Ikkβ: Inhibitor of nuclear kappa-B kinase subunit beta; Ikkγ: Inhibitor of nuclear kappa-B kinase subunit gamma; Ikkϵ: Inhibitor of nuclear kappa-B kinase subunit epsilon; IL-1β: Interleukin-1 beta; IL-8: Interleukin 8; IL-10: Interleukin-10; IL-12: Interleukin-12; IRAK1: Interleukin-1 receptor-associated kinase 1; IRAK2: Interleukin-1 receptor-associated kinase 2; IRAK4: Interleukin-1 receptor-associated kinase 4; IRF6: Interferon regulatory factor 6; JAK-STAT1-2: Janus kinase-Signal transducer and activator of transcription; JAK1: Janus kinase 1; JAK2: Janus kinase 2; JD: Johne’s disease; JNK1: c-Jun N-terminal kinase 1; JNK2: c-Jun N-terminal kinase 2; JNK3: c-Jun N-terminal kinase 3; kDa: kilo-Dalton; LAMP-3: Lysosome-associated membrane glycoprotein 3; M cell: Microfold Cell; ManLAM: Mannosylated lipoarabinomannan; MAP: Mycobacterium avium subsp. paratuberculosis; MAPK-p38: Mitogen-activated protein kinase p38; MEKK1-MKK1: Mitogen-activated protein kinase kinase kinase 1; MHCII: Majpr hisocompatability complex class-II; MKK2: Mitogen-activated protein kinase kinase kinase 2; MKK3: Mitogen-activated protein kinase kinase kinase 3; MKK4: Mitogen-activated protein kinase kinase kinase 4; MKK6: Mitogen-activated protein kinase kinase kinase 6; MLN: Mesenteric lymph nodes; MOI: Multiplicity of infection; MyD88: Myeloid differentiation primary response gene 88; NCBI-NR: National Center for Biotechnology Information – New RefSeq; NFκB p65: Nuclear factor kappa-light-chain-enhancer of activated B cells p65; NLRP3: NOD-like receptor family, pyrin domain containing 3; NRAMP1: Solute carrier family 11 member 1 (alternative name); NSF: N-ethyl-maleimide-sensitive factor (NSF); ODN: Oligodeoxynucleotides; PAK1: Serine-Threonine-protein kinase 1; PAMP: Pathogen associated molecular patterns; PBMC: Peripheral blood mononuclear cells; PCR: Polymerase Chain Reaction; PIP3: Phosphatidylinositol 3-phosphate; PKCα: Protein kinase C alpha; PKCβ: Protein kinase C beta; PRR: Pattern recognition receptor; PtpA: Protein tyrosine phosphatase A; PtpB: Protein tyrosine phosphatase B; Pyk2: Protein tyrosine kinase 2; Rab5: Ras-related protein 5; Rab7: Ras-related protein 7; Rac: Ras-related C3 botulinum toxin substrate; Raf: Serine-threonine-protein kinase; RelA: v-rel reticuloendotheliosis viral oncogene homolog A; RNI: reactive nitrogen intermediate; ROI: reactive oxygen intermediate; SapM: Secreted acid phosphatase; SC11A1: solute carrier family 11 member 1; Shc: Src homology 2 domain-containing; SHP-1: Src homology region 2 domain-containing phosphatase-1; SNAP: soluble NSF attachment proteins; SNARES: Vesicle and target membrane SNAP receptors; SOCS1: Suppressor of cytokine signaling proteins 1; SOCS3: Suppressor of cytokine signaling proteins 3; Src: Proto-oncogene tyrosine-protein kinase Src; STAT: Signal transducers and activators of transcription; TAB1: TGF-beta activated kinase 1; TAB2: TGF-beta activated kinase 2; TACO: Tryptophan aspartate-containing coat protein; TAK1: TGF-beta activated kinase 1; TBK1: TANK binding kinase 1; TGFb: Tumor growth factor beta; Th1: Type 1 helper T cell; Th2: Type 2 helper T cell; TLR1: Toll-like Receptor 1; TLR2: Toll-like Receptor 2; TLR9: Toll-like Receptor 9; TNFa: Tumor necrosis factor alpha; TNFR2: Tumor necrosis factor receptor superfamily member 1 beta; Tpl2: Tumor progression locus 2 protein kinase; TRAF6: TNF receptor-associated factor 6; V-ATPase: Vacuolar-type H^+^-ATPase; VPS33B: Vacuolar protein sorting-associated protein 33B.

## 10. Competing interests

The authors declare that they have no competing interests.

## 11. Authors’ contributions

All authors contributed content and editorial work to each section of this review. All authors read and approved the final manuscript.
